# Determinants of delay in malaria care-seeking behaviour for children 15 years and under in Bata district, Equatorial Guinea

**DOI:** 10.1186/s12936-016-1239-0

**Published:** 2016-03-31

**Authors:** Maria Romay-Barja, Jorge Cano, Policarpo Ncogo, Gloria Nseng, Maria A. Santana-Morales, Basilio Valladares, Matilde Riloha, Agustin Benito

**Affiliations:** Centro Nacional de Medicina Tropical, Instituto de Salud Carlos III, Madrid, Spain; Red de Investigación Colaborativa en Enfermedades Tropicales, RICET, Madrid, Spain; Faculty of Infectious and Tropical Diseases, London School of Hygiene and Tropical Medicine, London, UK; Centro de Referencia de Control de Endemias, Malabo, Equatorial Guinea; Ministerio de Salud y Bienestar Social, Malabo, Equatorial Guinea; Instituto Universitario de Enfermedades Tropicales y Salud Pública de Canarias, Tenerife, Spain

**Keywords:** Malaria, Care-seeking behaviour, Delay, Treatment, Bata district, Equatorial Guinea

## Abstract

**Background:**

Malaria remains a major cause of morbidity and mortality in children under 5 years of age in Equatorial Guinea. Early appropriate treatment can reduce progression of the illness to severe stages, thus reducing of mortality, morbidity and onward transmission. The factors that contribute to malaria treatment delay have not been studied previously in Equatorial Guinea. The objective of this study was to assess the determinants of delay in seeking malaria treatment for children in the Bata district, in mainland Equatorial Guinea.

**Methodology:**

A cross-sectional study was conducted in Bata district, in 2013, which involved 428 houses in 18 rural villages and 26 urban neighbourhoods. Household caregivers were identified in each house and asked about their knowledge of malaria and about the management of the last reported malaria episode in a child 15 years and younger under their care. Bivariate and multivariate statistical analyses were conducted to determine the relevance of socio-economic, geographical and behavioural factors on delays in care-seeking behaviour.

**Results:**

Nearly half of the children sought treatment at least 24 h after the onset of the symptoms. The median delay in seeking care was 2.8 days. Children from households with the highest socio-economic status were less likely to be delayed in seeking care than those from households with the lowest socio-economic status (OR 0.37, 95 % CI 0.19–0.72). Children that first received treatment at home, mainly paracetamol, were more than twice more likely to be delayed for seeking care, than children who did not first receive treatment at home (OR 2.36, 95 % CI 1.45–3.83). Children living in a distance >3 km from the nearest health facility were almost two times more likely to be delayed in seeking care than those living closer to a facility but with non significant association once adjusted for other variables (OR 1.75, 95 % CI 0.88–3.47).

**Conclusion:**

To decrease malaria morbidity and mortality in Bata district, efforts should be addressed to reduce household delays in seeking care. It is necessary to provide free access to effective malaria diagnosis and treatment, to reinforce malaria management at community level through community health workers and drug sellers and to increase awareness on the severity of malaria, the importance of early diagnosis and appropriate treatment.

## Background

Despite a strong decrease in incidence and mortality in the last decade, malaria continues to be a largest contributor to the burden of disease and premature death in many parts of sub-Saharan Africa. An estimated of 395,000 malaria deaths occur each year in the region, and 74 % of the deaths are in children under 5 years of age [1]. Most of these malaria deaths occur at home without receiving appropriate medical care and, when care is sought, it is often too late [2].

Early diagnosis and prompt treatment have been the cornerstones of successful malaria control. Early and appropriate treatment reduce illness progression to severe stages and, therefore, reduce mortality and morbidity rates, and onward transmission [3–6]. The risk of death from severe malaria is greatest within the first 24 h, but in most endemic countries, a long time passes before the patient receives care, delaying the start of appropriate anti-malarial treatment [7].

The World Health Organization established that early diagnosis and prompt treatment should occur within 24 h of the onset of malaria symptoms [8]. In 2010, the Roll Back Malaria partnership set a goal of ensuring that 80 % of malaria patients are diagnosed and treated with effective anti-malarial medicines within 1 day of the onset of illness, particularly those in the lowest two economic quintiles [9]. Most African countries are far from meeting this target [10].

Malaria is endemic in Equatorial Guinea with stable transmission [11] and remains a major cause of morbidity and mortality of children under five years of age [12]. A malaria control programme was introduced in 2007 in the mainland region, under the Equatorial Guinea Malaria Control Initiative (EGMCI). Case management was improved with the distribution of free artemisinin-based combination therapy to all health facilities, including the Community Health Workers (CHWs) trained to use both rapid diagnostic test (RDT) and artemisinin-based combination therapy (ACT). The initiative was largely funded by The Global Fund to Fight AIDS, Tuberculosis, and Malaria (GFATM), and it was implemented by the government of Equatorial Guinea, in collaboration with several international organizations. Unfortunately, with the withdrawal of the GFATM funding in 2011, the Initiative and its universal access to ACT were not maintained [13]. Despite the efforts of the EGMCI, malaria prevalence has remained high (41.2 %) in the Bata district in children under 15 years old [14]. Previous studies showed an important delay in seeking malaria treatment at the national level [15] and in the Bata district in particular [16], but information concerning determinants of this delay is insufficient in Equatorial Guinea.

Treatment-seeking behaviour is influenced by numerous factors, such as the caregiver’s level of education, the perception of the severity of the disease and the household's socio-economic status [2, 3]. Analysing the determinants of treatment delay may help strengthen interventions that aim to improve timely diagnosis and treatment of malaria. The purpose of this study was to assess the factors that affect delay in seeking treatment for malaria in children up to 15 years of age in the Bata district of Equatorial Guinea.

## Methods

### Study area and population

**Fig. 1:**
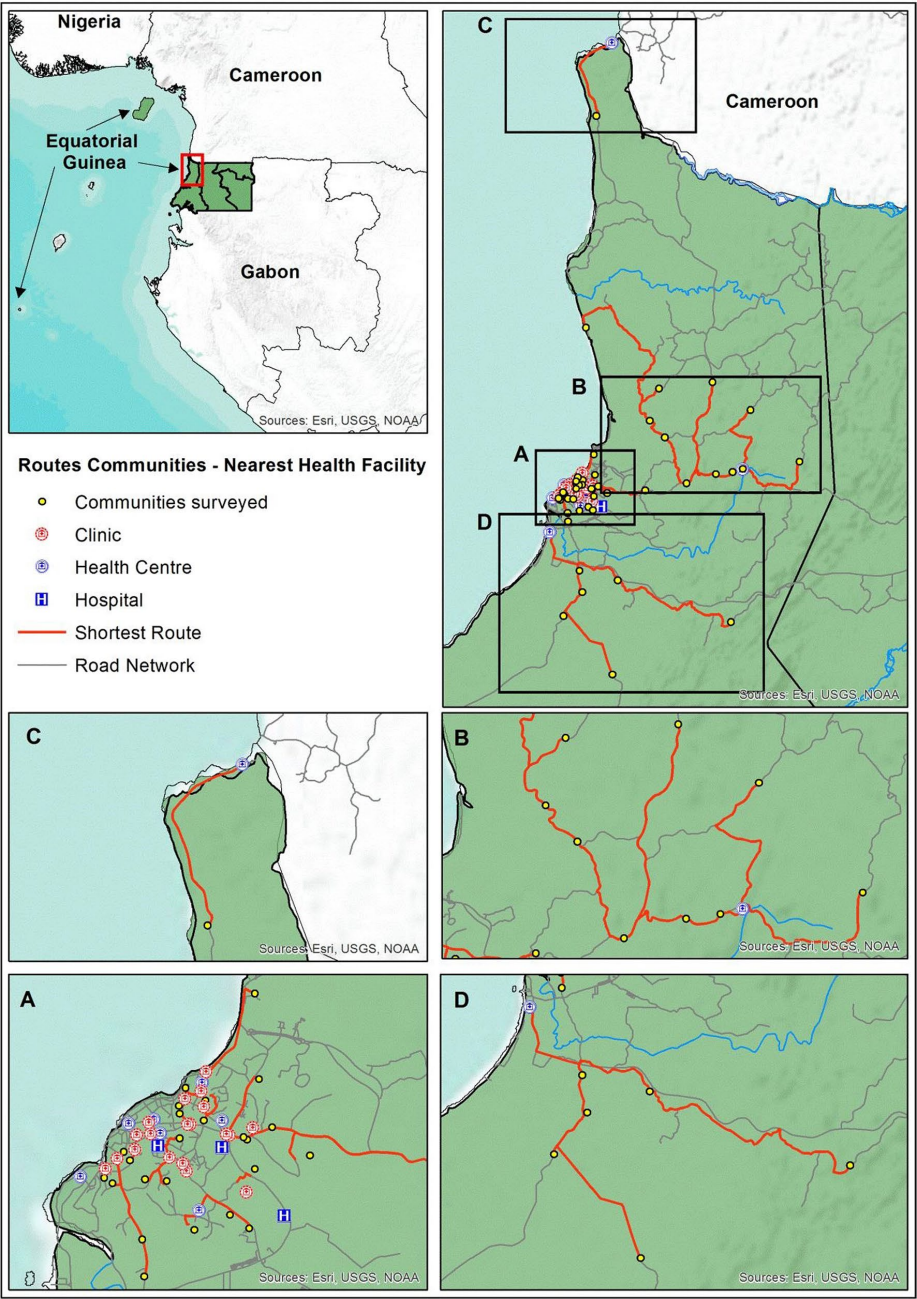
Map of public and private health facilities at Bata District, Equatorial Guinea. Inset maps shows details of the shortest route between communities and nearest health facilities, corresponding map **A** to urban communities (Bata quarters) and **B**, **C** and **D** to rural communities

The District of Bata, with a population of 244,264 inhabitants, is the largest district in the country, according to the latest national census [17]. The District’s public health facilities comprise a network of ten health centres, two rural and eight urban, and one regional hospital located in the city of Bata. There are also private health facilities in the Bata District, including two hospitals, and about 23 clinics, all in the urban area of Bata city (Fig. 1). About 156 pharmacies and other drug sellers are distributed throughout the rural and urban areas of the District. The first-line treatment for uncomplicated malaria in Equatorial Guinea is artesunate–amodiaquine (AS + AQ) and quinine is recommended for treating severe malaria.

A cross-sectional study was carried out in June–August 2013, in the Bata district, which is in the mainland region of Equatorial Guinea. The study was part of a project that aimed to provide baseline data on the prevalence of malaria, the molecular characteristics of *Plasmodium*, the vectors for malaria transmission in the area and to provide information about the malaria-related knowledge and practices of the target population.

### Sampling and data collection

A descriptive cross-sectional study was designed to determine whether caretakers were able to recognize malaria symptoms and to analyse the knowledge, attitudes and treatment practices of households related to the last reported malaria episode of children up to 15 years old in the district of Bata. Sampling was carried out with a multistage stratified cluster strategy. The strata were rural and urban settings, assuming that the 50 % of the population knew about malaria symptoms. First, rural villages and urban neighbourhoods were randomly selected with probability proportional to size to improve accuracy in sample design, resulting in a sampling size of 440 households. Second, households were randomly selected from an updated census from each cluster provided by the head of the village or neighbourhood. Other methodological aspects have previously been published [14, 16].

The household caregivers were identified in each house and asked about their knowledge of malaria and about their treatment-seeking behaviour for the last reported malaria episode in a child under their care. A structured questionnaire was designed and administered by trained field workers. Information about household social characteristics, about the caregiver's malaria associated knowledge and child symptoms, and about treatment and treatment timelines related to the last reported malaria episode in a child up to 15 years old was recorded.

A road network dataset was developed based on the OpenStreetMap (OSM) project [18] for the entire area of mainland Equatorial Guinea in order to estimate the shortest route from the villages and quarters to the nearest health facility, either public or private. An OSM layer with geographical feature was downloaded from the OSM project site, cropped to show the mainland region and converted into a compatible ArcGIS format using the OSM Network tools that are available in ArcGIS toolbox v10.2 (ESRI Inc., Redlands CA, USA). Finally, a road layer was transformed into a network dataset with connectivity across the entire network using rules for determining standard driving times. Standard driving rules were used to estimate impedance or cost of circulating across the road network as the use of vehicle, either particular or service transport (i.e. shared taxis), is widely extended and the most popular among Equatorial Guinea population above other means of transport such as bicycle or motorbikes [19]. In this way, it was possible to simulate driving on the road network and estimate the optimal routes between communities and health facilities. Impedance, namely the cost of moving across the network, was set in minutes, so that the optimal route was the one involving the shorted driving time. Travel time and total distance were calculated for each route in minutes and kilometres, respectively.

### Data analysis

The time elapsed between the onset of symptoms and the time when outside treatment was sought was defined as early if care was sought within 24 h and as delayed if more than 24 h passed between the onset of the symptoms and seeking treatment.

Using principal component analysis, a wealth indicator variable that served as a proxy for socio-economic status was created using household-owned assets, housing characteristics and the type of access to water and sanitation [20, 21]. The first principal component was considered as the summary measure of socio-economic status and subsequently divided into quintiles to assign houses to different wealth strata.

The multistage cluster sampling strategy used for this study was considered in the analysis by assessing the effect of the design on the variables of interest and applying it to the estimation of 95 % confidence intervals. Frequencies and percentages were used to summarize data and to assess factors related to delay, Student’s t test and χ^2^ tests for continuous and categorical variables, respectively, were performed. Comparisons for which p values were below 0.05 were considered significant. Mean and standard deviation or median and interquartile range were calculated for continuous variables that were or were not normally distributed, respectively. A bivariate analysis of the associations between the independent variables and delay was conducted using simple logistic regression. Independent variables that were significantly associated with delay at de p < 0.10 level, as well as child age, were included in the multivariable analysis. The absence of multicollinearity and interaction among independent variables were checked to be satisfied. Logistic regression models were obtained by using a manual backward stepwise procedure. The adjusted odds ratio (aOR) and 95 % confidence interval (95 % CI) were computed; p values less than or equal to 0.05 were considered statistically significant. Data analysis was performed using STATA software version 12.

### Ethics statement

This study was approved by the Ministry of Health and Social Welfare of Equatorial Guinea and the Ethics Committee of the Spanish National Health Institute, Carlos III (CEI PI 22_2013-v3). Written informed consent for participation in the study was obtained from the caregivers interviewed and from the heads of the households.

## Results

### Descriptive statistics

Of 428 caregivers who were interviewed about the last malaria episode in a child under their care, 62 only provided treatment at home, two provided no treatment at all and 28 did not remember the time that elapsed between the onset of the malaria symptoms and the time at which they sought treatment outside the home. Therefore, a total of 336 caregivers were included in this study. The caregivers interviewed had a median age of 31 years (IQR: 25–43; minimum: 15; maximum: 70), and most were female (98.5 %). In terms of educational background, 56.8 % of the caregivers had attended secondary school. The heads of households were mainly males (64.9 %) and an employment, whether in the formal or informal sector, was the main source of income (52.4 %). A total of 905 children were living in the 336 houses. There was a median of 1.3 children under 1 year of age per house (IQR: 1–1; minimum: 0; maximum: 6) and a median of four children aged 1–15 years per house (IQR 2–6; minimum: 0; maximum: 22). Most of the households (63.4 %) lived three kilometres or less from the nearest health facility and those who lived farther than three kilometres mainly lived in rural area (86.6 %).

**Fig. 2:**
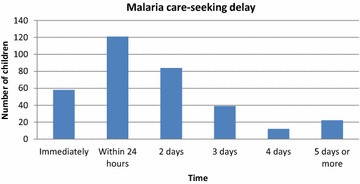
The time elapsed between malaria symptoms onset and seeking treatment for children in Bata district, Equatorial Guinea

Table 1 summarizes the characteristics of the households, the children and their reported malaria episode according to the delay in seeking treatment after the onset of symptoms. Most of the children were between one and five years of age (58.9 %). There were a slightly higher percentage of boys (53.65 %) than girls, and most of the children were taken to the health facility by their mothers (77.7 %). The two malaria symptoms that were most often identified in children by their caregivers were fever (86.0 %) and weakness (32.1 %), followed by nausea (20.8 %) and convulsions (11.3 %). Most of the children received treatment at home (67.9 %) before they were taken to a health facility. Paracetamol was the treatment that was most frequently administered at home (70.13 %). Only 9.96 % of the children who were first treated at home received anti-malarial medication, artemether being the anti-malarial that was administrated most frequently (3.45 %). Nearly half of the children (46.7 %) sought treatment at least 24 h after the onset of the symptoms (95 % CI 41.4–52.1 %). The median of delay in seeking care was 2.8 days with a range of 2–15 days. Figure 2 shows the distribution of children with reported malaria according the time that passed before the caregiver sought health care for them.

**Table 1:** Characteristics of children, their malaria episode and their households according to the delay in seeking care outside home

Children characteristics	Delay				p value*
	≤24 h		>24 h		
	n	%	n	%	
Age group (years)					
<1	28	52.8	25	47.2	
1–5	105	53.0	93	47.0	
>5	46	54.1	39	45.9	0.984
Sex of child					
Female	92	59.0	64	41.0	
Male	87	48.3	93	51.7	0.051
Area					
Urban	130	59.4	89	40.6	
Rural	49	41.9	68	58.1	0.002
Relationship of child to household head					
Child/grandchild	157	54.5	131	45.5	
Other	22	45.8	26	54.2	0.264
Signs and symptoms					
Fever (yes)	149	51.6	140	48.4	0.118
Weakness (yes)	65	60.2	43	39.8	0.081
Convulsions (yes)	25	65.8	13	34.2	0.101
Nausea (yes)	39	55.7	31	44.3	0.646
Headache (yes)	12	41.4	17	58.6	0.179
Treated at home first					
No	73	67.6	35	32.4	
Yes	106	46.5	122	53.5	<0.001
Who took the child for treatment?					
Father	6	42.9	8	57.1	
Mother	138	52.9	123	47.1	
Grandmother	24	60.0	16	40.0	
Father and mother	6	75.0	2	25.0	
Other	5	38.5	8	61.5	0.404
Children in the household					
Under 1 year old (yes)	123	51.0	118	49.0	0.191
From 1 to 15 years old (yes)	176	53.7	152	46.3	0.365
Household characteristics					
Sex of the head of household					
Female	66	55.9	52	44.1	
Male	113	51.8	105	48.2	0.472
Main source of money					
Agriculture	13	48.2	14	51.9	
Fishing	2	50.0	2	50.0	
Hunting	2	100.0	0	0.0	
Employee	94	53.4	82	46.6	
Business	57	50.9	55	49.1	
Other	11	73.3	4	26.7	0.449
Wealth quintiles					
Poorest	26	37.1	44	62.9	
Second	32	49.2	33	50.8	
Middle	38	53.5	33	46.5	
Fourth	37	58.7	26	41.3	
Richest	46	68.7	21	31.3	0.005
Distance to nearest health facility					
≤3km	129	60.6	84	39.4	
>3km	50	40.7	73	59.4	< 0.001
Caregiver characteristics					
Age					
15–34 years	102	51.8	95	48.2	
35 years or older	77	55.4	62	44.6	0.513
Sex					
Female	174	52.6	157	47.4	
Male	5	100.0	0	0.0	0.035
Ethnicity					
Fang	150	52.5	136	47.6	
Combe	17	56.7	13	43.3	
Bisio	3	100.0	0	0.0	
Ndowe	9	52.9	8	47.1	0.415
Education					
Primary school or less	69	48.9	72	51.1	
Secondary school and+	110	56.4	85	43.6	0.175
Marital status					
Widow	6	50.0	6	50.0	
Married	105	52.2	96	47.8	
Single	67	55.8	53	44.2	
Divorced	1	33.3	2	66.7	0.817
Relationship of caregiver to the household head					
Wife	75	48.7	79	51.3	
Mother	2	66.7	1	33.3	
Self	41	57.8	30	42.3	
Daughter	42	59.2	29	40.9	
Sister	12	54.6	10	45.5	
Others	7	46.7	8	53.3	0.601
Considered malaria to be a problem					
No	27	45.0	33	55.0	
Yes	152	55.1	124	44.9	0.156
Knew mosquitos cause malaria					
No	66	47.1	74	52.9	
Yes	113	57.7	83	42.4	0.057
Someone in the house has died of malaria before					
No	168	54.37	141	45.63	
Yes	11	40.74	16	59.26	0.173

* χ^2^ test

### Factors affecting delay in seeking care

**Fig. 3:**
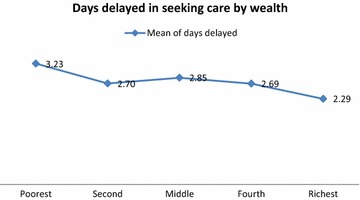
The delay between malaria symptoms onset and seeking treatment for children in the Bata district, according to household wealth (divided into quintiles)

According to the bivariate analysis, delay in seeking treatment was significantly higher in rural areas (58.1 %), in boys (51.7 %) and in children that first received treatment at home (52.3 %). Delay in seeking treatment outside home was also significantly higher in those who lived more than three kilometres from the nearest health centre (59.4 %), and in children whose caregivers did not know that malaria is transmitted by a mosquito (52.9 %). Delay was most frequent in the poorest households (62.9 %) and showed a significant trend: the higher the wealth level, the shorter the delay (p < 0.0001). Figure 3 shows the mean of delay in days according to household socio-economic status.

In the multiple logistic regression analysis (Table 2), those children that received treatment at home first were 2.4 times more likely to be taken for care later than those who did not after adjusting for other variables (95 % CI 1.45–3.83). Low socio-economic status was also a significant factor for delay. The richest households were 2.7 times more likely to seek early treatment than the poorest once adjusted for other variables. Children who lived more than three kilometres away from the nearest health facility were almost two times more likely to show a delay in seeking care than those who lived closer, with non significant association (95 % CI 0.88–3.47) once adjusted for other variables.

**Table 2:** Factors associated with delay in seeking treatment out of home determined by multiple logistic regression

	Unadjusted OR (95 % CI)	Adjusted OR (95 % CI)
Sex of child		
Female		
Male	1.54 (0.99–2.39)	1.43 (0.88–2.33)
Area		
Urban		
Rural	2.03 (1.23–3.34)	0.83 (0.41–1.68)
Treated at home first		
No		
Yes	2.40 (1.48–3.91)	2.36 (1.45–3.83)
Wealth quintile*		
Poorest		
Second	0.61 (0.23–1.26)	0.63 (0.30–1.29)
Middle	0.51 (0.24–1.11)	0.59 (0.27–1.31)
Fourth	0.42 (0.20–0.88)	0.52 (0.23–1.15)
Richest	0.27 (0.15–0.48)	0.37 (0.19–0.72)
Distance to nearest health facility		
≤3km		
>3km	2.24 (1.38–3.65)	1.75 (0.88–3.47)
Knew mosquitos cause malaria		
No		
Yes	0.66 (0.42–1.01)	0.77 (0.48–1.22)

* p value <0.0001 for the F test for trend

## Discussion

Delay in seeking treatment for malaria is an important risk factor for severe complications in children. This remains a serious problem in many countries in sub-Saharan Africa, including Equatorial Guinea. This study found that being treated at home first and the socioeconomic level of the household were determinants of delay in seeking-care for children with malaria in the Bata district.

Almost half of the children in the study, between 41.4 and 52.1 % of children in Bata district, were not taken to seek health care for at least 24 h after the onset of symptoms. This delay is similar to that found in a previous national study [15] and in other studies in the region [22–24].

Treating malaria at home was the first option for most caregivers in the Bata district [16]. This behaviour is common in most African areas with high malaria transmission rates, even in areas in which biomedical and health services are widely available and accessible [25, 26]. Receiving home treatment first, mainly paracetamol, was one of the most important factors associated with delay in seeking care in Bata district and it may contribute to the development of severe complications related to the illness [5]. Management of illness by community health workers improves timely access to treatment and is associated with a reduction in malaria infections [27]. Unfortunately, CHWs have not had access to diagnostic tools or to treatments in Bata district since 2011, due to financial constraints and continuous stock shortages [16].

Although most heads of households in the Bata district recognized the main symptoms of malaria, this knowledge was not associated with early treatment as caregivers seem to perceive their child’s illness as being mild [28]. In many places, knowledge about malaria symptoms is not associated with seeking timely treatment for children [23, 29]. Consistent with this, the present study found that Bata district caregivers who reported having a death in the family that was caused by malaria did not seek treatment for their child earlier than other caregivers. These data indicates that caregivers in Bata district may have a low perception of illness severity that could be causing delay in seeking adequate treatment.

Occupational status of the heads of households and the educational level of the caregivers are not always associated with delay in seeking treatment for malaria [29] and this was found in Bata district as well. In addition, the age of the child was not a determinant for delay in seeking care, in contrast with the association found in other studies [30]. Furthermore, the sex of the child is not usually associated with delay in malaria diagnosis and treatment [31, 32], but one study in a Nairobi slum area showed that boys were more likely to get early diagnosis and treatment of malaria [33]. In Bata district, the sex of the children was not significantly associated with delay, but boys were taken later than girls to seek treatment out of home.

In the Bata district, households that were three kilometres or less from the nearest health facility tended to seek malaria treatment for children earlier. This finding is consistent with many other studies [29, 34] showing that mothers who have to travel less than three kilometres are more likely to seek early treatment for their children with malaria compared to those who had to travel farther. Most of these children lived in urban Bata. In rural areas, the most accessible healthcare providers for caregivers are some drug sellers, the CHWs and two government health posts poorly provided, while in urban areas there is a wider offer of public and private health facilities. When a caregiver must pay for transportation, those with limited resources may not seek timely treatment for a child who is thought to have malaria because they may need to find money for transport first [29, 30].

Affordability is a major barrier to malaria treatment and health care throughout sub-Saharan Africa [22] and a number of studies have reported an association between socioeconomic status and delay in seeking care [24, 34, 35]. In Bata district, there is an important association between household’s socio-economic status and delay in seeking care. The trend was clear: households at the highest socioeconomic level were almost three times less likely to delay seeking care compared to the poorest families. In Bata district, the delay in seeking treatment for children with malaria depended much more on household wealth than on the distance to the nearest health facility. Caretakers reported paying a median of 18.24 € for the last malaria treatment for their child [16], a cost very difficult to afford for the poorest families, regardless of where they live.

This study has some limitations. Firstly, the treatment-seeking behaviour registered was based on reported malaria thus some cases may not have been malaria. However, this would not have changed the behaviour of caregivers because they thought it was malaria and proceeded accordingly. Secondly, due to sample size, some associations may not show significant in multivariable logistic regression such as the association between distance to a health centre and delay in seeking care.

## Conclusions

Low socio-economic status and provide treatment at home before seeking care were factors significantly associated with delay in seeking care for children with reported malaria in the Bata district. The Equatorial Guinea Malaria Control Initiative should focus its strategy in the mainland region on guaranteeing free access to malaria treatment to families, especially to the poorest families. Reinforcing malaria management at home and providing rapid diagnostic tools and adequate treatment to Community Health Workers and drug sellers have proven to be crucial for timely access to quality malaria treatment. Programmes that increase the community awareness of the seriousness of malaria and the importance of early diagnosis and proper treatment are also needed.
